# Metabologenomics analysis of *Pseudomonas* sp. So3.2b, an Antarctic strain with bioactivity against *Rhizoctonia solani*

**DOI:** 10.3389/fmicb.2023.1187321

**Published:** 2023-05-04

**Authors:** Naydja Moralles Maimone, Mario Cezar Pozza Junior, Lucianne Ferreira Paes de Oliveira, Dorian Rojas-Villalta, Simone Possedente de Lira, Leticia Barrientos, Kattia Núñez-Montero

**Affiliations:** ^1^'Luiz de Queiroz' Superior College of Agriculture, Department of Math, Chemistry, and Statistics, University of São Paulo, Piracicaba, São Paulo, Brazil; ^2^Biotechnology Research Center, Department of Biology, Instituto Tecnológico de Costa Rica, Cartago, Costa Rica; ^3^Extreme Environments Biotechnology Lab, Center of Excellence in Translational Medicine, Universidad de La Frontera, Temuco, Chile; ^4^Facultad Ciencias de la Salud, Instituto de Ciencias Biomédicas, Universidad Autónoma de Chile, Temuco, Chile

**Keywords:** bioactivity, OSMAC, molecular networking, secondary metabolites, genomics, biosynthetic gene cluster, bioprospecting

## Abstract

**Introduction:**

Phytopathogenic fungi are a considerable concern for agriculture, as they can threaten the productivity of several crops worldwide. Meanwhile, natural microbial products are acknowledged to play an important role in modern agriculture as they comprehend a safer alternative to synthetic pesticides. Bacterial strains from underexplored environments are a promising source of bioactive metabolites.

**Methods:**

We applied the OSMAC (One Strain, Many Compounds) cultivation approach, in vitro bioassays, and metabolo-genomics analyses to investigate the biochemical potential of *Pseudomonas* sp. So3.2b, a strain isolated from Antarctica. Crude extracts from OSMAC were analyzed through HPLC-QTOF-MS/MS, molecular networking, and annotation. The antifungal potential of the extracts was confirmed against *Rhizoctonia solani* strains. Moreover, the whole-genome sequence was studied for biosynthetic gene clusters (BGCs) identification and phylogenetic comparison.

**Results and Discussion:**

Molecular networking revealed that metabolite synthesis has growth media specificity, and it was reflected in bioassays results against R. solani. Bananamides, rhamnolipids, and butenolides-like molecules were annotated from the metabolome, and chemical novelty was also suggested by several unidentified compounds. Additionally, genome mining confirmed a wide variety of BGCs present in this strain, with low to no similarity with known molecules. An NRPS-encoding BGC was identified as responsible for producing the banamides-like molecules, while phylogenetic analysis demonstrated a close relationship with other rhizosphere bacteria. Therefore, by combining -omics approaches and *in vitro* bioassays, our study demonstrates that *Pseudomonas* sp. So3.2b has potential application to agriculture as a source of bioactive metabolites.

## 1. Introduction

Phytopathogenic fungi can lead to substantial crop losses, as many are causative agents of plant diseases that are difficult to manage. *Rhizoctonia solani* is a necrotrophic soil fungus that can harm several commercial crops such as soybean, maize, and other essential plants from Amaranthaceae, Araceae, Asteraceae, Brassicaceae, Fabaceae, Linaceae, Malvaceae, Poaceae, Rubiaceae, and Solanaceae families (Ajayi-Oyetunde and Bradley, 2018). Currently, *R. solani* management can be partially performed by agrochemicals. Still, this approach is costly and is often related to environmental risks like soil contamination, the extermination of non-targeted populations of life forms, and even the impact on human health (Tahmidur Rahman et al., 2020). From this perspective, using natural products isolated from antagonistic microorganisms for pest control is usually an advantageous alternative to using harmful pesticides in the field crops. Natural products are currently quoted as promising tools for sustainable crop management (Pirttilä et al., 2021; Palmieri et al., 2022).

However, several challenges must be overcome for a microbe-derived bioactive compound to be discovered and become a product (Li et al., 2019). For instance, chemical redundancy in biodiscovery programs has presented itself as an obstacle (Chandra Mohana et al., 2018; Qin et al., 2022). The natural products community has used different methodological strategies to overcome these difficulties and make biodiscovery more efficient. One of these is to search for bioactive compounds in strains from extreme environments, as these places constitute underexplored areas and, over time, extremophile microorganisms developed their ways to survive and occupy niches under drastic conditions of temperature, salinity, humidity, pH, radiation, and many other stresses (Yadav, 2021). Therefore, the natural history of these environments has led to the evolution of microbial groups with unique properties, as in those locations, their chemical potential could be enhanced naturally (Maurya et al., 2020). For example, several *Pseudomonas* strains from extreme environments such as polar regions led to isolating important novel bioactive compounds like diketopiperazines, phenazine alkaloids, pigments, and exopolysaccharides (Jayatilake et al., 1996; Carrión et al., 2015; Styczynski et al., 2022).

Another approach for biodiscovery programs consists of diversifying culture conditions, such as culture media composition, temperature, pH values, and other factors. The so-called OSMAC (One Strain, Many Compounds) strategies are based on the fact that even minor variations in culture conditions can promote the synthesis of several different secondary metabolites by the same strain (Bode et al., 2002). OSMAC experiments may be carried out by evaluating culture conditions individually to find the most exciting results, assisting the optimization of natural compound production through complete experimental planning with statistical analysis (Romano et al., 2018).

On the other hand, -omics techniques can also aid in avoiding redundancy and rediscovery of natural products from microbial cultures. Through metabolomics, it is possible to explore the metabolic information of biological samples, annotate known compounds, recognize the structural differences in the compounds and their analogs by computational methods, and guide the selection of metabolites for isolation (Atanasov et al., 2021). Besides, the genomic era has increased the possibilities for discovering new natural products. Studies have demonstrated that the genes responsible for producing specialized bacterial metabolites often lie together within a genome as biosynthetic gene clusters (BGCs) (Kenshole et al., 2021). The automated BGC finding in genome sequences has significantly contributed to the mining of microbial genomes for natural product discovery, also helping to predict their synthesis pathways, regulation, transport, and molecular structure in some cases (Kenshole et al., 2021). Hence, the combination of metabolomics and genomics allows the establishment of links between BGCs and the metabolites, increasing the chances of predicting bioactivity and molecular structures of potentially novel compounds (Leão et al., 2022).

The genus *Pseudomonas,* one of the most abundant groups of bacteria dwelling in the soil and plant rhizosphere, is known due to its broad lines of action in agricultural needs, including the production of several enzymes and metabolites skilled in the suppression of phytopathogens (Nadeem et al., 2016). Currently, some *Pseudomonas*-based bioproducts are available on the market to implement the control of essential plant diseases agents like *Rhizoctonia solani, Botrytis cinerea, Colletotrichum graminicola, Erwinia amylovora, Fusarium oxisporum, Michridichium nivale, Sclerotinea homeocarpa*, and others with equal importance (Korshunova et al., 2021). However, those are strain-based products. Comparatively, there are only a few commercial products based on *Pseudomonas*-derived metabolites, despite the large number of natural products isolated from them (Jahanshah et al., 2019; Mevers et al., 2019). Also, some *Pseudomonas* strains and other groups prospected for commercial formulations can cause opportunistic infections, requiring more sophisticated characterizations for safe use (Kumari et al., 2022). In this sense, it is essential to demonstrate the bioactivity of the strain’s extracts, as their natural products may represent better options to be applied in the field than the strains *per se*. We had previously isolated a bacterial strain (So3.2b) from Antarctic soil. We identified it through complete genome analysis as a *Pseudomonas* specie, with an Average Nucleotide Identity (ANI) of 99.33 and 99.07% with the species *P. shahriarae* and *P. fluorescens*, respectively (Núñez-Montero et al., 2023). Here, we describe the chemical potential of this strain by using OSMAC cultivation approaches, metabologenomics analysis, and elucidating its bioactivity against five strains of *R. solani* from different plant sources.

## 2. Materials and methods

### 2.1. Bacterial culture and metabolites extraction

The acquisition of extracts for LC–MS/MS analysis was achieved by using a 24-well microbioreactor system (Applikon Biotechnology Inc., Nederland). For that, a colony of the bacterial strain was inoculated in 1.5 mL of four broth media including M2 (Mannitol 40.0 g/l, Maltose 40.0 g/l, Yeast extract 10.0 g/l, K_2_HPO_4_ 2.0 g/l, MgSO_4_·7H_2_O 0.5 g/l and FeSO_4_·7H_2_O 0.01 g/l), IMA (Yeast extract 4 g/l, Malt extract 10 g/l, Glucose 4 g/l, Mannitol 40 g/l), YES (Sucrose 150 g/l, Yeast extract 20 g/l, MgSO_4_·7H_2_O 0.5 g/l, ZnSO_4_·7H_2_O 0.01 g/l and CuSO_4_·5H_2_O 0.005 g/l) and CGA (Glycerol 30 g/l, Peptone 2 g/l, K_2_HPO_4_ 1 g/l, NaCl 1 g/l, MgSO_4_·7H_2_O 0.5 g/l and 5 mL of trace solution containing CaCl_2_·2H_2_O 3 g/l, MnSO_4_ 0.2 g/l, ZnCl_2_ 0.1 g/l, CuSO_4_·5H_2_O 0.025 g/l, Na_2_B_4_O_7_·10H_2_O 0.02 g/l, CoCl_2_ 0.004 g/l and (NH_4_)_6_Mo_7_O_24_·4H_2_O 0.01 g/l). Extraction of secondary metabolites was performed using ethyl acetate after 7 days of incubation at 15°C and 190 rpm. We also obtained extracts from un-inoculated wells for each media to be used as blank controls. For this study, a single well for treatments and blanks was extracted. Then, to acquire the necessary number of extracts for antifungal assays, we performed a scaled-up cultivation process. Basically, after three days of incubation in TSA medium (16°C), the bacterial strain inoculum was set in 0.85% sodium chloride solution to 1×10^7^ CFU (McFarland standard), and 150 μL were inoculated to Erlenmeyer’s with 150 mL of the same culture media described previously and incubated in the same conditions. Liquid–liquid extraction was performed with ethyl acetate, and the resulting extracts were dried on a rotary evaporator and kept under refrigeration until use on bioassays.

### 2.2. Detection of secondary metabolites by LC-QTOF-MS/MS under different culture media

An *in-situ* extraction of each culture was done by adding 2 mL of ethyl acetate to each well of the previously grown cultures on the microbioreactor system. After incubation for 60 min at 190 rpm at room temperature, the organic phase containing secondary metabolites was collected, dried under N_2_ airflow, and resuspended in 20 μL of methanol to generate the analytes for LC-QTOF-MS/MS and to conduct antimicrobial assays. Aliquots of each extract (1 μL) were analyzed by LC-QTOF-MS/MS, in a Agilent 1290 Infinity II UHPLC coupled to an Agilent 6545 LC/QTOF mass spectrometer with an orthogonal electrospray ionization (ESI) interface (Agilent Technologies, Waldbronn, Germany); using a Zorbax C8 RRHD 1.8 μm (2.1 × 50 mm) column, elution gradient of 2.50 min at 0.417 mL/min from isocratic 90% H2O/MeCN (acetonitrile) to 100% MeCN (with isocratic 0.1% formic acid modifier). MS/MS analysis was performed on the same instrument for ions detected in the full scan at an intensity above 1,000 counts at 10 scans/s, with an isolation width of ∼4 m/z using a fixed collision energy of 20 eV and a maximum of three selected precursors per cycle. Samples were injected (5 μL) using an autosampler refrigerated at 4°C.

### 2.3. Mass spectrometry data processing and analysis

Data were converted from raw to mzXML format with MSConvert (Chambers et al., 2012) and uploaded to the GNPS platform (Wang et al., 2016) for further analysis. A molecular network was created with filtered data (MS2 fragment ions within ±17 Da of the precursor *m/z* were removed). To window filter MS2 spectra, only the top 6 fragment ions in the ±50 Da window throughout the range were chosen. The precursor ion mass tolerance was set to 0.02 Da, and an MS2 fragment ion tolerance of 0.02 Da. The network was created by filtering edges to a cosine score above 0.7 and more than six matched peaks, and boundaries between two nodes were kept in the network if and only if each of the nodes appeared in each other’s respective top 10 most similar nodes. The spectra in the network were searched against GNPS’ spectral libraries. The library spectra were filtered in the same manner as the input data. All matches kept between network spectra and library spectra were required to have a score above 0.7 and at least six matched peaks. The network can be accessed by the link: https://gnps.ucsd.edu/ProteoSAFe/status.jsp?task=879d66d4fb9c44cc83d9fb4031646223. Results were visualized on Cytoscape software (Shannon et al., 2003). A PCoA plot (based on Bray–Curtis dissimilarity metrics) was used to graphically demonstrate the distance measure between each of the treatments based on their overlapping molecules, and this plot was visualized with the EMPeror Qiime2 plugin (Bolyen et al., 2019). To improve the knowledge about the chemical potential of our bacterial strain by increasing the metabolite annotation rates, we also performed *in silico* analysis with the NAP tool (da Silva et al., 2018) which allowed us to achieve MSI level 3 annotations (Sumner et al., 2007). For that, we constructed a library with compounds isolated from *Pseudomonas* spp. retrieved from the Natural Products Atlas database (van Santen et al., 2019). Then, we performed the analysis considering [M + H]^+^, [M + Na]^+^ and [M + K]^+^ as possible adducts for the hits and 15 ppm as a tolerance for the accuracy of the exact mass of the candidate structures. The results can be accessed by the following link: https://proteomics2.ucsd.edu/ProteoSAFe/status.jsp?task=92a813db795c433ba7426e63b46b413e.

### 2.4. Antifungal bioassays

The activity of extracts obtained from the *Pseudomonas* sp. strain cultivated on different culture media was evaluated against *Rhizoctonia solani* strains by the mycelial growth inhibition test (Rios et al., 1988), with some modifications. Briefly, after resuspension in sterilized dimethyl sulfoxide (DMSO) and distilled water (1:9), the extracts were added to autoclaved PDA medium shortly before its solidification and gently stirred to assure homogenization and to reach a final concentration of 250 μg mL^−1^ of extracts on medium before pouring it into Petri dishes Plugs of 5 mm of radius containing fresh fungal mycelium were added to the center of the treatment plates, which were incubated at 28°C for as long as necessary for the phytopathogens in each negative control plate to take up all available space for growth. As a positive control, we used the Maxim^®^ fungicide, with its active ingredients diluted at the same concentration as the extracts, and as a negative control, we used the 10% DMSO solution. Each treatment was evaluated in triplicates against all the fungi strains. The percentage of fungal growth inhibition was measured with ImageJ software, the means were calculated and then compared by Tukey test with a script in the R language. The phytopathogenic *R. solani* strains used in this study are CMES 1861 (isolated from *Glycine max*), CMAA 1592 (isolated from *Ocimum basilicum*), CMAA 1417 (isolated from *Cichorium endivia*), CMAA 1588 (isolated from *Solanum tuberosum*) and CMAA 1589 (isolated from *Origanum vulgare*). Those were kindly provided by EMBRAPA - Soybean and EMBRAPA - Environment.

### 2.5. Genomic mining of biosynthetic gene cluster and chemical-genomic annotation comparison

The complete genome sequence of the *Pseudomonas* sp. strain So3.2b was previously obtained and reported (Núñez-Montero et al., 2023), which is available at National Center for Biotechnological Information (NCBI) by the accession number CP080494.1. Biosynthetic gene clusters were identified through antiSMASH v6.1.1 (Blin et al., 2021) with “relaxed” detection strictness, for annotation of well-defined and low similarity clusters without incurring into false positives. Chemically annotated compounds were compared with genomic annotations to find similarities. NPA026209 and NPA026208 annotated as possible bananamides D and F, respectively, showed genetic structural similarity to a Non-Ribosomal Peptide Synthases (NRPS) cluster in region 7 (Supplementary Table S1). To confirm this similarity, protein-coding sequences of the *Pseudomonas* sp. strain So3.2b and biosynthetic gene cluster of bananamides D and F from *Pseudomonas* sp. strain COW3 (GenBank accession: MN480426.1) (Omoboye et al., 2019) were obtained (consulted 11/3/2022). Synthetic blocks were identified through local colinearity regions using progressive mauve alignment tool with Mauve v2.4.0.

### 2.6. Genomic phylogenetic analysis

A phylogenetic analysis was carried out for a better understanding of the evolutionary relationship between So3.2b strain and its biosynthetic potential. Phylogenetic distances were determined by constructing of core proteome of the available complete representative genomes of the NCBI (entered 11/10/2022) for *Pseudomonas* genus (×100) plus the bananamide-producer COW3 strain (total: 102 genomes, including our Antarctic strain). Core proteome were obtained with M1CR0B1AL1Z3R (Avram et al., 2019)^1^ using the parameters of 0.01 maximum E-value and 80.0% minimum identity in all compared genomes. Briefly, the tool extracts all ORFs from all genomes using Prodigal, detects homologous genes (all against all) using MMSEQS2 and then clusters them using MCL and uses MAFFT to reconstruct an amino acid (AA) multiple sequence alignment (Avram et al., 2019). Once aligned, M1CR0B1AL1Z3R reverse translates each AA alignment to get the corresponding codon alignment. The phylogenetic tree was constructed with this proteome alignment using maximum likelihood algorithm with RAxML (Stamatakis, 2014). The resulting tree was visualized using iTOl v6.6 (Letunic and Bork, 2021).

## 3. Results and discussion

### 3.1. Chemical analysis and metabolites annotation

**Figure 1:**
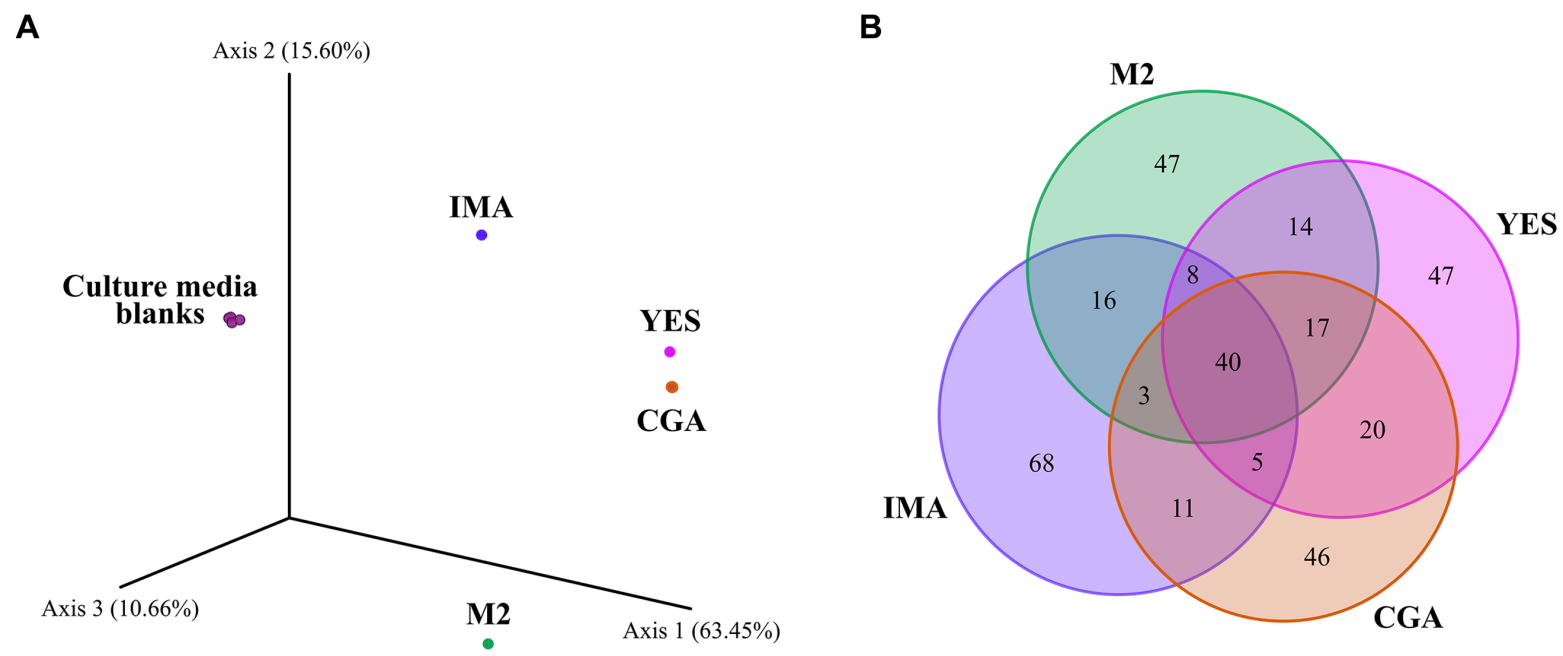
Bray-Curtis-based PCoA highlighting the chemical dissimilarity between extracts of *Pseudomonas* sp. cultivated on different culture media **(A)**. Venn diagram with the total number of features detected on the extracts **(B)**.

To study the effect of nutrient sources on the production of specialized metabolites by *Pseudomonas* sp. So3.2b, the strain was grown on four nutrient-rich media with different carbon sources to stimulate antimicrobial production. OSMAC approaches have allowed the discovery of new microbial compounds through easily adaptable changes, such as altering the carbon and nitrogen source, the concentration of nutrients, pH, or temperature (Li et al., 2013; Sha and Meng, 2016; Zhang et al., 2017). It is supposed to mimic natural environmental changes and can promote the expression of silent biosynthetic gene clusters inducing the microorganism to synthesize secondary metabolites with diverse scaffolds (Schwarz et al., 2021). Furthermore, alterations in the C/N ratio considerably affect the pH of the culture media through the formation of acids and organic bases (Pan et al., 2019). In Figure 1, the production of specific metabolites in each culture media can be observed, with the IMA medium having the highest amount of exclusively detected features and IMA and M2 having the most dissimilarity compared to the others. In this case, when the culture medium has a mixture of rapidly-assimilation and slowly-assimilation carbon sources, the former is preferentially used to produce cells and compounds of primary metabolism. After this faster assimilation, the second carbon source can be used to create specialized metabolites (Ruiz et al., 2010).

It is known that the presence of two or more carbon sources in the growing environment has a relevant impact on growth. Regulation of the sequential use of carbon sources in microorganisms is governed by carbon catabolite repression (CCR) and has a significant effect on many other cellular functions. The assembly of regulatory networks coordinates the differential expression of genes under limiting and non-limiting carbon source conditions. These mechanisms promote the use of nutrients that support high growth rates and consequently restrict the expression of non-essential genes for growth, avoiding high metabolic costs. Although knowledge about CCR is ancient, the mechanism for the formation of secondary metabolites in the *Pseudomonas* genus is not yet well established (Shahid et al., 2018; Ruiz-Villafán et al., 2022).

**Figure 2:**
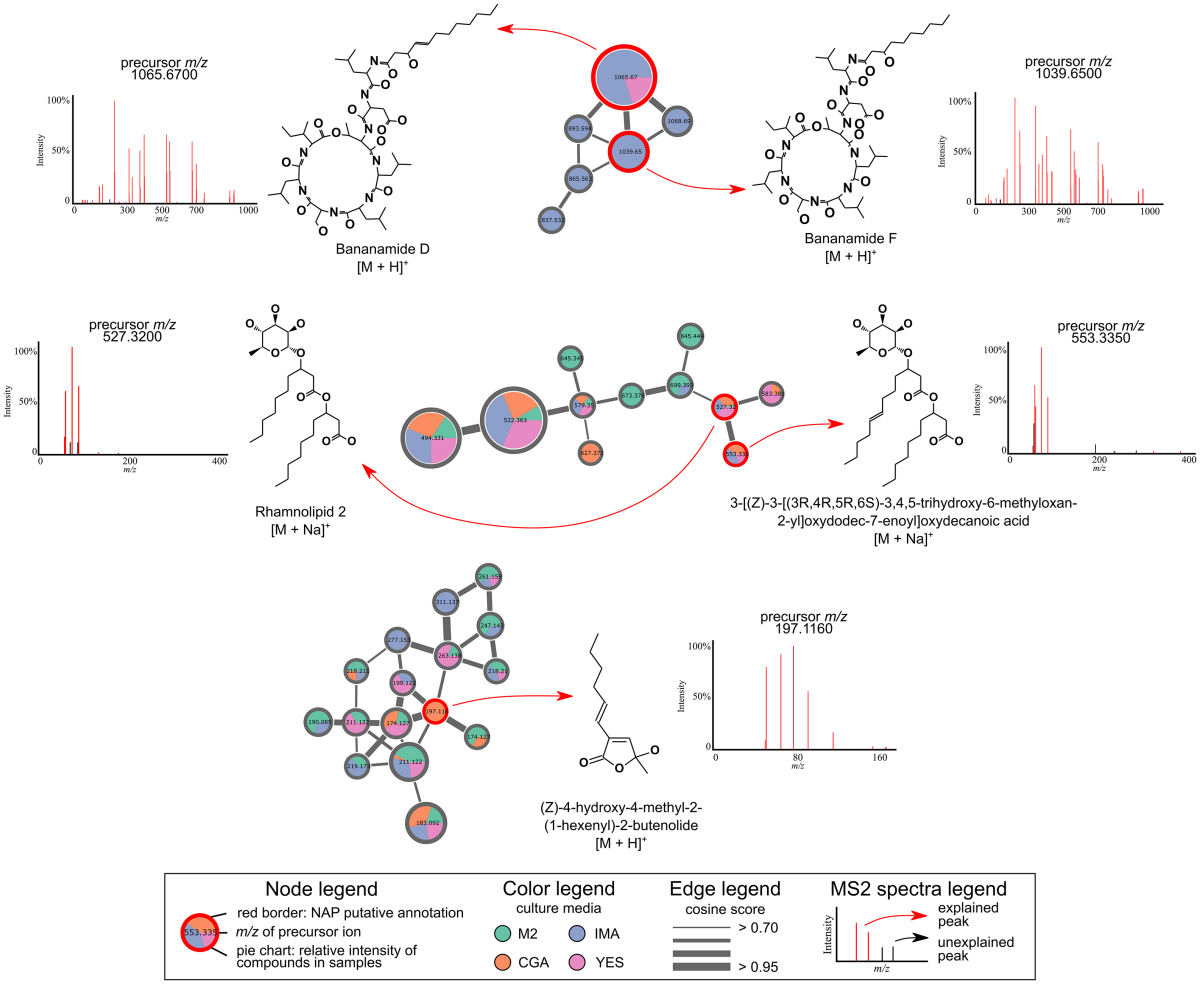
Molecular families generated by GNPS molecular networking with level 3 annotations (MSI) obtained in the NAP analysis.

Using culture media with different nutrient sources provided the synthesis of exclusive compounds in each culture condition in our study. Mapping culture media information on molecular networks made it easier to visualize these changes (Supplementary Figure S1). These approaches combined have already led to the discovery of several new microbial compounds (Crüsemann et al., 2017; Zang et al., 2020; Han et al., 2022). The molecular network itself presents a strategy used mainly in the natural products field, as comparing and linking spectra with similar fragmentation patterns make it easier to recognize the chemical space that comprehends a set of samples, to determine the presence of analogs or new derivatives of known compounds and to perform annotation propagation within molecular families (Atanasov et al., 2021). The Global Natural Products Social (GNPS) molecular networking platform also provides users with a spectral library of thousands of compounds, and experimental spectra can be automatically searched along the analyses (Wang et al., 2016; Aron et al., 2020). However, despite the increasing number of free-to-access spectral information available, annotating mass spectrometry data is still challenging. An excellent strategy to improve annotation rates is to use *in silico* tools and take advantage of structural libraries, which are much larger than spectral libraries. Here, we applied the NAP tool to promote *in silico* annotations of our spectral data based on a structural library composed only of compounds produced by *Pseudomonas* strains retrieved from the Natural Products Atlas platform. The putative annotation for (Z)-4-hydroxy-4-methyl-2-(1-hexenyl)-2-butenolide is observed only in the CGA medium, besides several related compounds were also detected in all the other media (Figure 2). In addition, rhamnolipids were also found in all culture conditions, but specific features were exclusive to M2 and CGA media. The bananamides’ cluster features were mainly detected in the IMA media, with bananamide F also found in YES media (Figure 2).

In this work, the putatively annotated bananamides were bananamide D (detected *m/z:* 1065.670, [M + H]^+^, molecular formula: C_53_H_92_N_8_O_14_) and F (detected *m/z* 1039.650, [M + H]^+^, molecular formula C_51_H_90_N_8_O_14_). The Δ*m/z:* 26.02 Da between those annotations is consistent with the observed C_2_H_2_ difference on the compounds. Bananamides are cyclic lipopeptides (CLPs) commonly found in the *Pseudomonas* genus and produced by NRPS biosynthetic gene clusters (Nguyen et al., 2016). CLPs are biosurfactants, and their mode of action generally consists of the penetration of the plasma membrane, creating pores that dysregulate ion fluxes and finally lead to the death of the cell (Bender et al., 1999). The first bananamide-like structure was isolated and elucidated from *Pseudomonas granadensis* (Geudens and Martins, 2018). Subsequently, another bananamide-type compound was isolated and characterized by Nguyen et al. (2016) from a strain of *Pseudomonas fluorescens* isolated from the banana rhizoplane. These compounds have great potential for biotechnological use as an antibiotic, antifungals, and other antimicrobial activities (Cameotra and Makkar, 2004; Geudens and Martins, 2018). Within their functionality, antifungal properties have been presented by bananamides D-F against phytopathogens *Pythium myriotylum* and *Pyricularia oryzae* (Omoboye et al., 2019).

We also were able to annotate the (Z)-4-hydroxy-4-methyl-2-(1-hexenyl)-2-butenolide compound (detected *m/z* 197.116, [M + H]^+^, molecular formula C_11_H_16_O_3_), which belongs to the butyrolactone class. This compound was isolated and characterized from *Pseudomonas aureofaciens* strain 63-28 and had antimicrobial activity against *Pythium ultimum*, *Rhizoctonia solani,* and *Phytophthora cryptogea* (Pascale et al., 1997). Butyrolactones are not exclusively detected on *Pseudomonas*, being also produced by actinobacteria (Franco et al., 1991) and, mainly, by fungi strains (Chen et al., 2015; Zhang et al., 2020; Tilvi et al., 2022), with several bioactivities reported. Due to their antimicrobial potential, synthetically obtained butyrolactones have also been designed and have demonstrated promising results to be applied as antiviral and antifungal agents in agriculture (He et al., 2022; Wu et al., 2022).

Finally, rhamnolipids are widely known biosurfactants, belonging to the class of glycolipid biosurfactants, mainly produced by *Pseudomonas aeruginosa*, the first one being reported since the 60s (Edwards and Hayashi, 1965). As it has been studied for decades, this molecule has several attributed activities, including anticancer (Rahimi et al., 2019), antifungal (Vatsa et al., 2010; Carrión et al., 2015; Sha and Meng, 2016) and antibiofilm activity (Silva et al., 2017). Due to their high biodegradability and non-toxicity, rhamnolipids have gained attention in different sectors, especially health and the environment (Thakur et al., 2021). Regarding the *in silico* annotation we got rhamnolipid 2 (detected *m/z* 527.320, [M + Na]^+^, molecular formula C_26_H_48_O_9_), which has shown potential application in soil remediation (Kristoffersen et al., 2018). The other annotated rhamnolipid (detected *m/z* 553.335, [M + Na]^+^, molecular formula C_28_H_50_O_9_), also isolated from an Antarctic *Pseudomonas* strain, has a bactericidal effect against a set of pathogens (Tedesco et al., 2016). In a previous study, OSMAC approaches have led to the discovery of novel rhamnolipids from another Antarctic *Pseudomonas* strain (Kristoffersen et al., 2018).

It is worth noticing that all the annotated compounds in this work are part of molecular families with multiple non-annotated adduct ions that represent compounds structurally similar to the rhamnolipids, bananamides or the (Z)-4-hydroxy-4-methyl-2-(1-hexenyl)-2-butenolide. On molecular network analysis, within a molecular family with annotations, those unknown features can represent different adducts from the same known compounds (in positive mode for example, a Δ*m/z* of approximately 23 Da between two nodes can be indicative of a [M + Na]^+^ adduct, while a Δ*m/z* of approximately 39 Da can be indicative of a [M + K]^+^ adduct) or adducts from known compounds for which there are any available data on the databases used in the research, but might also characterize potentially new analogs for the annotated compounds (easy to detected examples are analogs with a methylation difference, Δ*m/z* of approximately 14 Da, and an oxygen loss, Δ*m/z* of approximately 16 Da) (Aron et al., 2020). We should also mention that besides the importance and usefulness of the annotation tools to enhance our comprehension upon the complex mixtures that characterize natural extracts, further isolation and structural elucidation are required for identification of metabolites, and bioassays based on pure compounds should be performed to confirm their bioactivities. *In silico* tools, like the one we used in this work, are usually more assertive on predicting molecular classes than the compounds for a given feature (da Silva et al., 2018), and so additional care must be taken by researchers using them. In this work, our approach used a database composed only of *Pseudomonas*-derived compounds in order to diminish false annotation rates, and we also inspected the predicted peaks to the substructures they were assigned for on the annotations. Even so, exploratory studies like this undoubtedly can take advantage of using such tools to highlight the chemical potential of promising strains (Crüsemann et al., 2017; Bauermeister et al., 2018).

### 3.2. Bioactivities of extracts against *Rhizoctonia solani* strains

The crude extracts obtained from *Pseudomonas* sp. So3.2b culture in four different media was evaluated against five *R. solani* strains from diverse plant sources (Supplementary Figure S2). Most extracts inhibited at least one of the tested strains, except for the *R. solani* strain CMAA1592 which was not inhibited by any extract (Table 1). By comparing the bioassay results, the M2 culture medium presented the broadest potential for producing inhibitory compounds, reducing the growth of strains CMAA1417, CMAA1589, and CMAA1588 by 35,17, 60.45%, and 90,57%, respectively. The extract from IMA medium was also bioactive against more than one *R. solani* strain (CMES1861 - 67.67%, CMAA1588 - 43.74%). As shown in Figure 1A, those were the extracts with higher dissimilarity compared to the others evaluated in this work in the Bray-Curtis-based PCoA. Notably, the IMA medium produces a greater diversity of compounds, demonstrated through the greater amount of unique ions detected (Figure 1B), and was responsible for the production of the most bananamide-like molecular networks (Figure 2), where most ions were only produced when So3.2b was cultivated in this medium. The promising inhibitory effect of this crude extract remains expected, considering the well-demonstrated antifungal activity of bananamides and bananamides-like molecules from other *Pseudomonas* sp. strains isolated from plant rhizospheres (Omoboye et al., 2019).

**Table 1:** Inhibition percentage of *R. solani* strains by extracts of *Pseudomonas* sp. So3.2b and the commercial fungicide Maxim^®^ (250 μg mL^−1^).

Treatment	*R. solani* CMES1861	*R. solani* CMAA1588	*R. solani* CMAA1589	*R. solani* CMAA1417	*R. solani* CMAA1592
YES	–	21.78% ± 1.14 e	–	–	–
CGA	–	24.94% ± 0.41 d	–	–	–
M2	–	90.57% ± 0.69 b	60.45% ± 3.5 b	35.17% ± 9.69 b	-
IMA	67.67% ± 1.25 b	43.74% ± 1.34 c	–	–	–
Maxim^®^	100% ± 0.00 a	100% ± 0.00 a	100% ± 0.00 a	100% ± 0.00 a	100% ± 0.00 a

In the same column, values assigned the same letter are not significantly different (*p* < 0.05) according to Tukey’s test.

On the other hand, some rhamnolipids-related molecules were exclusively produced in M2 medium. Since those molecules are known to have bioactivity against other microbes, they are likely partially responsible for the antifungal activity observed in this work and the improved activity compared to other culture mediums, as previously studies have stated (Sha and Meng, 2016; Jishma et al., 2021). Additionally, the presence of rhamnolipids producer strains of *Pseudomonas* sp. in the rhizosphere of agriculturally relevant plants and their antifungal properties incur in the idea of a protective role against phytopathogens (Jishma et al., 2021). This result confirms the antifungal potential of Antarctic *Pseudomonas* strain So3.2b and its chemical production potential under OSMAC approach. Notably, the observed activity was obtained from the crude extract. Then, a stronger antifungal activity might be obtained from purified fractions of one or more compounds isolated from this strains for agricultural management.

As *Pseudomonas* strains are ubiquitous to various environments, they are commonly isolated (Mielko et al., 2019), and have been notably used as biocontrol agents in agriculture (Dimkić et al., 2022; Guzmán-Guzmán and Santoyo, 2022), which suggests their chemical capacity. There are reports on literature on the potential of their metabolites, both volatile and non-volatile, against phytopathogenic microorganisms including *R. solani* (Sasirekha and Srividya, 2016; Wang et al., 2021). Phenazines (Shanmugaiah et al., 2010), furanones (Paulitz et al., 2000), pyrroles (Howell, 1979; Cartwright et al., 1995), and cyclic lipopeptides (Hua and Höfte, 2015; Oni et al., 2020) are among the main compounds responsible for the activity related specifically against this fungal pathogen. Regarding to the cyclic lipopeptides, it is interesting to bring up the results reported by Nielsen et al. (2000), who detected and isolated a tensin compound from extracts of a *P. fluorescens* strain and tried to apply an OSMAC approach to study tensin production and search for other bioactive compounds produced on different culture media. Their study demonstrated tensin quantitative variation related to changes on nutrients sources but did not detect the production of other bioactive compounds in response to those changes. In our study, we report a differential production of the annotated bananamides according to the culture media used. Siderophores produced by *Pseudomonas* may also be related to antifungal activity against *R. solani* (Sasirekha and Srividya, 2016). Although we did not annotate any siderophore from our extracts through spectral and *in silico* libraries searches, nonetheless a BGC encoding pyoverdine-like compound was detected on genome annotation (Table 2).

**Table 2:** Biosynthetic gene cluster of *Pseudomonas sp.* So3.2b annotated by antiSMASH with previously reported antifungal activity.

Region	Type	Most similar known cluster	Similarity (%)	From	To	Reference to previous antifungal activity report
1	RiPP-like	–	–	174,367	184,064	Saravanan et al. (2021)
2	NRPS-like	Nematophin	12	374,268	403,055	Zhang et al. (2019)
3	Arylpolyene	APE Vf	35	677,437	721,011	Abdel-Mageed et al. (2020)
4	RiPP-like	–	–	1,619,296	1,630,177	Saravanan et al. (2021)
6	NRPS	Pyoverdin	17	2,353,769	2,406,285	Dutta et al. (2020) and Zhao et al. (2021)
7	NRPS	Pyoverdin	20	2,455,913	2,549,388	Dutta et al. (2020) and Zhao et al. (2021)
10	Acyl amino acids	A33853	8	3,554,000	3,614,929	Wang et al. (2022)

RiPP, Ribosomally synthesized and Post-translationally modified Peptide; NRPS, Non-Ribosomal Peptide Synthetase; -, Non found.

Microbial prospecting efforts in the Antarctic environment have also long led to the isolation of several psychrophilic *Pseudomonas* strains (Witter, 1961), including novel species (Jang et al., 2020; Nováková et al., 2020), and plenty of those have been screened for a plethora of applications (Ruan et al., 2005; Stallwood et al., 2005; Yarzábal et al., 2018). To the best of our knowledge, no previous studies reported antifungal activity from Antarctic-derived *Pseudomonas* against *R. solani* specifically, but we do have reports on antifungal potential of some strains against other phytopathogenic fungi (Poblete-Morales et al., 2020). Psychrophilic and psychrotolerant bioactive microorganisms are expected to represent good opportunities for the development of new biopesticides and biofertilizers for agriculture in regions of cold climate conditions (Yarzábal et al., 2018; Torracchi et al., 2020). But beyond that, their metabolome cannot be unconsidered, as it is widely accepted that they represent promising strains for natural products discovery (Núñez-Montero and Barrientos, 2018), as also revealed by genome analysis data (including this work) (Lee et al., 2017; Poblete-Morales et al., 2020). As demonstrated by our bioassay results, metabolites produced by *Pseudomonas* from Antarctica can lead to phytopathogen control even for fungal strains isolated from tropical climates. Also, by using the OSMAC approach we could better understand this strain’s bioactivity profile. Considering both bioassay results and metabolomics analysis under the extracts, we hypothesize that more than one compound or compound class can be related to the antifungal activity reported, depending on the culture medium evaluated. Although crude extracts can be used on exploratory research such as this, leading to the assessment of chemical and bioactivity profiles of talented microorganisms (Cumsille et al., 2017; Bauermeister et al., 2018; Sabido et al., 2021), further approaches such as fractionation of extracts and the use of metabolomics tools such as Bioactivity-based molecular networks can lead to the detection of the compounds responsible for the activity and its isolation (Nothias et al., 2018).

### 3.3. Characterization of identified biosynthetic gene clusters

*Pseudomonas* sp. strain So3.2b has twelve different BGCs in its genome principally associated with ribosomally synthesized and post-translationally modified peptides (RiPP), NRPS, and N-acetylglutaminylglutamine amide dipeptide (NAGGN), as well as other chemicals compounds like arylpolyene, betalactone, butyrolactone, hserlactone and a redox cofactor (Supplementary Table S1). Little to no similarity is noted between the identified molecules and previously reported clusters. The highest similarity percentage is the arylpolyene (35%) to a known APE Vf. These results suggest the idea of new molecules in this bacterial strain that could be related to the antifungal activity earlier discussed. Certain RiPP, arylpolyene, NRPS, and acyl amino acids have reported inhibitory effects against fungi (Table 2), therefore, may be related to the bioactivity of the strain.

**Figure 3:**
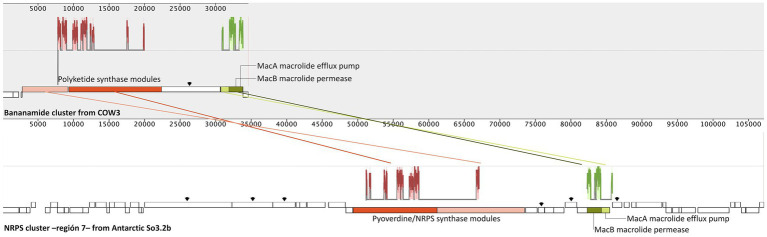
Syntenic comparison by alignment and identification of local collinearity blocks (LCBs) between the biosynthetic gene clusters from bananamide DF of *Pseudomonas* sp. COW3 (accession MN480426.1) and homologous regions of Non-Ribosomal Peptide Synthases (NRPS) cluster in the region 7 of the Antarctic *Pseudomonas* sp. So3.2b. Gene graphics represent each cluster with homologous LCBs color matching. Similar plots for LBCs are shown on the top of genes graphics. Homologous genes are labeled with its annotation description, additional biosynthetic genes are pointed by arrows and other genes are presented as white boxes.

To relate the genetic biosynthetic content with predicted molecules from chemical analysis, we conducted a comparison between each of the BGCs of the Antarctic strain against the gene cluster encoding the indicated molecules. No related genes were found with the production of rhamnolipids or (Z)-4-hydroxy-4-methyl-2-(1-hexenyl)-2-butenolide. Nonetheless, the latter is likely to be produced by an unknown butyrolactone gene cluster found on the genome (BGC region 9, Supplementary Table S1). Meanwhile, bananamides D-G of the *Pseudomonas* sp. COW3 strain (Omoboye et al., 2019) showed the most significant similarity with the NRPS type gene cluster of region 7, which comprises five biosynthetic genes, seven regulatory genes, and 11 transport-related genes. Moreover, similar adenylation, condensation, and thiolation domains, transcriptional regulator genes, outer membrane lipoprotein genes, and efflux proteins of the bananamides-producer cluster are also present in So3.2b strain, indicating that this could be the biosynthetic cluster responsible for producing bananamide-like compounds (Supplementary Table S2; Omoboye et al., 2019). Bananamides compounds were characterized in *Pseudomonas fluorescens* strain BW11P2 (later reclassified as *P. bananamidigenes*) isolated from banana (*Musa* sp.) rhizosphere in Sri Lanka and named bananamides A-C (Nguyen et al., 2016). As our Antarctic strain is close to being considered a *P. fluorescens* strain, regarding average nucleotide identity comparison value above the same species indicator (>95%) (Núñez-Montero et al., 2023). It is reported that BGCs encoding cyclic lipopeptides in *Pseudomonas* have a significant degree of synteny, and the natural products synthesized from them often possess a high structural similarity (Christiansen et al., 2020). Therefore, the similarity between the chemically annotated and genomic compared molecules was confirmed through alignment of local collinear blocks between both COW3, and So3.2b strains clusters (Figure 3).

Bananamides D and F were chemically annotated for our Antarctic strain, and we confirm their genetic similarity sharing most of the biosynthetic genes of COW3 bananamide cluster (Figure 3). Hence, our results suggest that bananamide-like compounds are probably encoded by the NRPS cluster of region 7 of the Antarctic So3.2b strain. The bananamide-like compounds detected in this study are likely associated with its bioactive antifungal potential since the NRPS genetic cluster responsible for its production also contains multiple antibiotic resistance genes (e.g., macrolide resistance *macAB*), an observation in antibiotic producer microorganisms as a mechanism to avoid suicide by their own produced molecules (Almabruk et al., 2018). Even though our results suggest region 7 might be responsible for the bananamide-like compound detected in *Pseudomonas* So3.2b, this BGC also contains multiple other biosynthetic genes (Supplementary Table S2; Figure 3) that might produce several different combinations molecularly and structurally similar to bananamides, such as those analogs adduct ions detected on Figure 2 (Chevrette et al., 2020).

Nonetheless, Antarctic *Pseudomonas* sp. So3.2b additionally has six BGCs associated with molecules of previously reported antimicrobial properties with low or no similarity to known molecules (highest similarity 35% of region 3 to an APE Vf, arylpolyene). Then, genomic and metabolomic pieces of evidence suggest that the Antarctic *Pseudomonas* So3.2b is a good source microorganism for the biodiscovery of novel antifungal products in the light of the non-traditional environmental isolation bioprospecting (Sekurova et al., 2019; Matos De Opitz and Sass, 2020). A similar approach to the one we used in this work was applied by Wang et al. (2020) to detect antifungal compounds produced by *P. aeruginosa*, where they compared metabolomics and genomics data of a bioactive (isolated from housefly gut) and an inactive strain (isolated from a wastewater treatment plant). Other similar approaches have been proposed to other Antarctic microorganisms of the proteobacteria phylum for natural product discovery (Núñez-Montero et al., 2020, 2022), and for several other bacteria from diverse environments (Maansson et al., 2016; Paulus et al., 2017; Tareen et al., 2022). Metabologenomics have been applied to fungi at a lower scale as the presence of introns in their genome makes gene prediction even more complex, but successful examples are available in literature (Gonçalves et al., 2022; Hebra et al., 2022), and some tools are being developed to facilitate metabologenomics analysis of data from fungi strains (Caesar et al., 2023). It demonstrates the understanding consensus of the scientific community that linking metabolome and genome information can indeed accelerate natural products discovery. This approach has also been eased by the lowering costs of genome sequencing and mass spectrometry analysis on recent years (Van Der Hooft et al., 2020) and by the development of several tools that aim to automate the integration of both data types (Soldatou et al., 2019), besides manual correlation is still largely used (Van Der Hooft et al., 2020).

### 3.4. Phylogenetic analysis

A phylogenic analysis based on the core proteome was carried out using the complete genomes of the *Pseudomonas* genus. The resulting tree showed the greatest vicinity with *P. shahriarae* (putative *P. fluorescens*), *P. yamanorum* and *P. veronii*, all isolated from agricultural soil samples (Supplementary Figure S3). Our Antarctic strain is distant from the bananamides-producer *P. botevensis* COW3, therefore in their evolutionary history, othertraits, related to environmental adaptation might conferred a considerable phylogenetic distance in spite of sharing similar bananamides D and F biosynthetic clusters. Hence, it is likely that this similar biosynthetic pathways were independently acquired/evolved for each species. Also, this similar BGCs might be more related to an acquired characteristic to cope with specific environment than a vertical genetic evolution [such as for some core secondary metabolism (Chevrette et al., 2020)]. The Antarctic environment where strain So3.2b was isolated is considered poly-extreme due to the cold weather, dry conditions, and high contamination regarding agrochemical pollutants, heavy metals, and antibiotics possibly transported by anthropogenic activities, water, air or volcanoes eruptions (Zreda-Gostynska et al., 1997; Bhardwaj et al., 2018; Chu et al., 2019; Vergara et al., 2019; Hwengwere et al., 2022). Also, studies have shown that non-indigenous bacteria introduced in the Antarctic continent led to an increase in genetic diversity, including antibiotic resistance genes, intensifying the evolutionary and selective pressure toward survival adaptations (Cowan et al., 2011; Hwengwere et al., 2022). These conditions restrict the survival and growth of microorganisms and confers the selective environmental pressure that affects gene flow mechanisms related to evolution, such as horizontal gene transfer events (HGT), leading to similar biosynthetic gene clusters independently evolved, including NRPS clusters (Chevrette et al., 2020).

The secondary metabolism developing through the recycling and repurposing of previous existing biosynthetic machinery– mediated by evolutive events such as horizontal gene transfer, or duplication is solid in antimicrobial compounds where an intense selective pressure is present (Chevrette et al., 2020). These ideas correlate with our results, where bananamide-like compounds were detected but showed substantial genetic differences on the known clusters responsible for their production, including multiple additional regulations, transport, and biosynthetic genes in the Antarctic *Pseudomonas* So3.2b (Figure 3). Additionally, genetic clusters tend to recruit genetic elements from phylogenetically distant clusters due to their wide range of substrates, positively affecting the selective pressure (Medema et al., 2014). Therefore, we propose our Antarctic strain could have evolved the bananamides-producer cluster independently through evolution events pressured by the poly-extreme environmental conditions of Antarctica. This explains the considerable phylogenetic distance between the bananamide producer COW3 and our So3.2b strains, regardless of the presence and synthesis of similar bananamides compounds with considerable BGCs differences.

## 4. Conclusion

Here we show genomic and metabolomic evidence about the potential of *Pseudomonas* sp. strain So3.2b metabolites for controlling *R. solani* strains from different crops. Thus, Antarctic microorganisms can be considered sources of bioactive compounds for agriculture in tropical and subtropical locations. In our chemical analysis, we highlight the detection of diverse chemical classes in bacterial extracts. We also detected spectral features to which no annotations could be designated, even for some of those found within molecular families with putatively identified compounds, which can be considered an indicator of chemical novelty. Our Antarctic strain also presented a wide variety of biosynthetic gene clusters with little to no similarity with known molecules, most with previously reported antimicrobial activity. Within these, region 7 encoding a NRPS is considered similar to the chemically annotated banamides-like molecules but containing multiple other biosynthetic genes. The phylogenetic analysis showed a great distance between the first bananamides D and F producer strain and our Antarctic strain, suggesting an independent evolution or acquisition of this BGC, which might be related to its response to the extreme environmental conditions from Antarctica. Therefore, both genomics and metabolomics analyses support that this Antarctic strain is a promising source for exploring bioactive compounds and application of these against phytopathogenic fungi. Our results also emphasize the importance of culture media variation in screening studies for natural product discovery (e.g., OSMAC approach).

## Data availability statement

The datasets presented in this study can be found in online repositories. The names of the repository/repositories and accession number(s) can be found in the article/Supplementary material.

## Author contributions

KN-M, DR-V, NM, MJ, and LO: conceptualization, methodology, validation, formal analysis, data curation, writing—original draft preparation, writing—review and editing, and visualization. SL and LB: conceptualization, supervision, project administration, and funding acquisition. All authors contributed to the article and approved the submitted version.

## Funding

This research was funded by grants from the Agencia Nacional de Investigacion y Desarrollo de Chile (ANID) - FONDECYT-1210563 and 11230475, the Instituto Antártico Chileno (INACH), INACH DG_01-19; Network for Extreme Environments Research (NEXER), NXR17-0003; Instituto Tecnológico de Costa Rica project 5402-1510-1035, CONICYT–PFCHA/Doctorado Nacional/2017-21170263 for KN-M, DR-V, and LB, by the Coordenação de Aperfeiçoamento de Pessoal de Nível Superior – Brasil (CAPES) – Finance Code 001 (scholarship to NM, LO, and MJ), by São Paulo State Funding Agency, Brazil (FAPESP research grant 2019/17721-9 to Roberto Gomes de Souza Berlinck and scholarship 2022/01529-4 to NM) and the National Council for Scientific and Technological Development (CNPq scholarship 142260/2020-4 to LO and 141501/2020-0 to MJ).

## Conflict of interest

The authors declare that the research was conducted in the absence of any commercial or financial relationships that could be construed as a potential conflict of interest.

## Publisher’s note

All claims expressed in this article are solely those of the authors and do not necessarily represent those of their affiliated organizations, or those of the publisher, the editors and the reviewers. Any product that may be evaluated in this article, or claim that may be made by its manufacturer, is not guaranteed or endorsed by the publisher.

## Footnotes

^1^https://microbializer.tau.ac.il/
