# Supplementary material for: Metabologenomics analysis of Pseudomonas sp. So3.2b, an Antarctic strain with bioactivity against Rhizoctonia solani
Source: Front Microbiol. 2023 May 4;14:1187321. doi: 10.3389/fmicb.2023.1187321 (PMC10192879; doi:10.3389/fmicb.2023.1187321)
Supplement: Supplementary file 1 [file Data_Sheet_1.docx]

Supplementary Material

Metabologenomics analysis of *Pseudomonas sp.* So3.2b, an Antarctic strain with bioactivity against *Rhizoctonia solani*

Naydja Moralles Maimone, Mario Cesar Pozza Junior, Lucianne Ferreira Paes de Oliveira, Dorian Rojas-Villalta, Simone Possedente de Lira, Leticia Barrientos*, Kattia Núñez-Montero*.

*** Correspondence:** Leticia Barrientos: [leticia.barrientos@ufrontera.cl](mailto:email@uni.edu) and Kattia Núñez-Montero: [k.nunez03@ufromail.cl](mailto:k.nunez03@ufromail.cl)

**
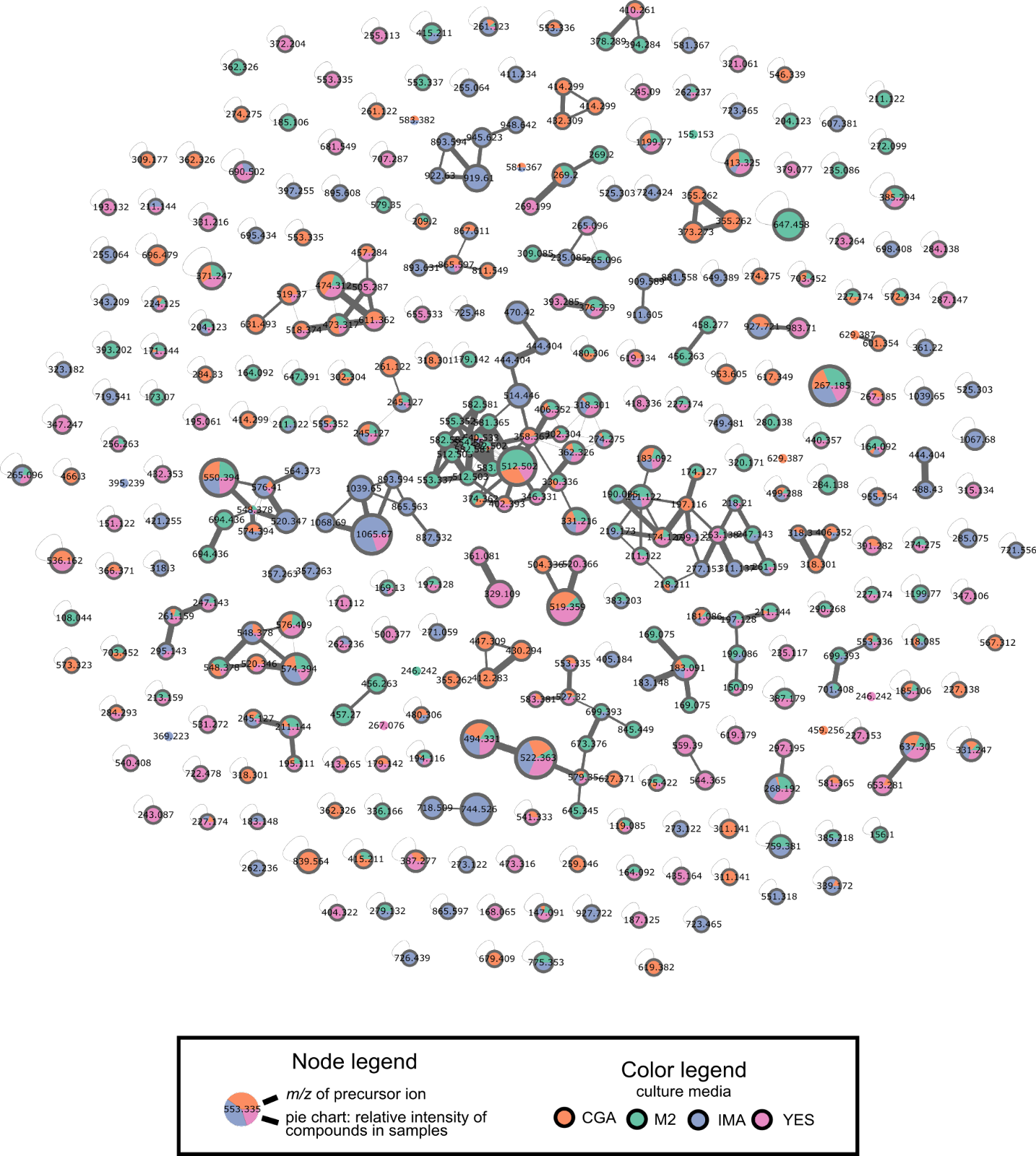
**

**Supplementary figure 1.** Molecular network of *Pseudomonas sp.* So3.2b cultivate under different culture media.

**Supplementary table 1.** Biosynthetic Gene Cluster (BGC) annotated in *Pseudomonas sp.* So3.2b strain genome through antiSMASH v6.1.1 tool, in ‘relaxed’ detection strictness.

| **Region** | **Type** | **Most similar known cluster** | **Similarity (%)** | **from** | **to** |
| --- | --- | --- | --- | --- | --- |
| **1** | RiPP-like |  |  | 174367 | 184064 |
| **2** | NRPS-like | Nematophin | 12 | 374268 | 403055 |
| **3** | Arylpolyene | APE Vf | 35 | 677437 | 721011 |
| **4** | RiPP-like |  |  | 1619296 | 1630177 |
| **5** | NAGGN |  |  | 2204456 | 2219315 |
| **6** | NRPS | Pyoverdin | 17 | 2353769 | 2406285 |
| **7** | NRPS | Pyoverdin | 20 | 2455913 | 2549388 |
| **8** | Hserlactone |  |  | 2772460 | 2793038 |
| **9** | Butyrolactone |  |  | 3406893 | 3420309 |
| **10** | Acyl amino acids | A33853 | 8 | 3554000 | 3614929 |
| **11** | Betalactone | Fengycin | 13 | 3869451 | 3892657 |
| **12** | Redox cofactor | Lankacidin C | 13 | 5792523 | 5814670 |

RiPP: Ribosomally synthesized and Post-translationally modified Peptide. NRPS: Non-Ribosomal Peptide Synthetase. NAGGN: N-acetylglutaminylglutamine amide dipeptide.


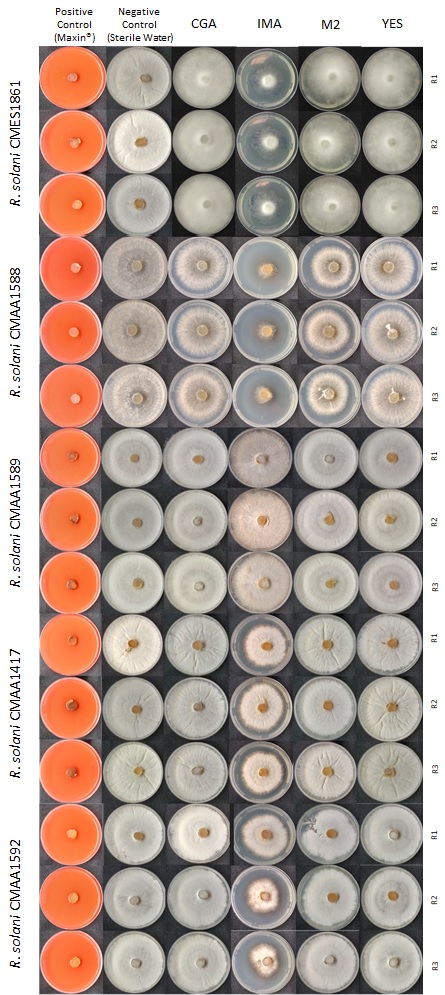


**Supplementary figure 2**. Antifungal bioassay applying *Pseudomonas* sp. So3.2b extract (250 µg ml^-1^) with five phytopathogenic strains of *Rhizoctonia* *solani*. The *R. solani* strains are CMES 1861 (isolated from *Glycine max*), CMAA 1592 (*Ocimum basilicum*), CMAA 1417 (*Cichorium endivia*), CMAA 1588 (*Solanum tuberosum*) and CMAA 1589 (*Origanum vulgare*).

**Supplementary table 2.** Functional genomic comparison of common genes present in bananamides’ biosynthetic cluster of *Pseudomonas sp.* COW3 and *Pseudomonas sp.* So3.2b.

| **Function** | ***Pseudomonas sp.* COW3** | ***Pseudomonas sp.* So3.2b** |
| --- | --- | --- |
| **Transport-related genes** | RND efflux system, outer membrane lipoprotein | RND efflux system, outer membrane lipoprotein |
| **Biosynthetic gene** | Non-ribosomal peptide synthetase | Condensation domain containing protein |
| **Biosynthetic gene** | Non-ribosomal peptide synthetase | Condensation domain containing protein |
| **Transport-related genes** | Macrolide efflux protein *MacA* | ABC transporter related protein |
| **Transport-related genes** | Macrolide efflux protein *MacB* | Resistance *MacB*, ABC transporter related protein |
| **Regulatory gene** | *LuxR* family transcriptional regulator | *LuxR* family DNA binding response regulator |


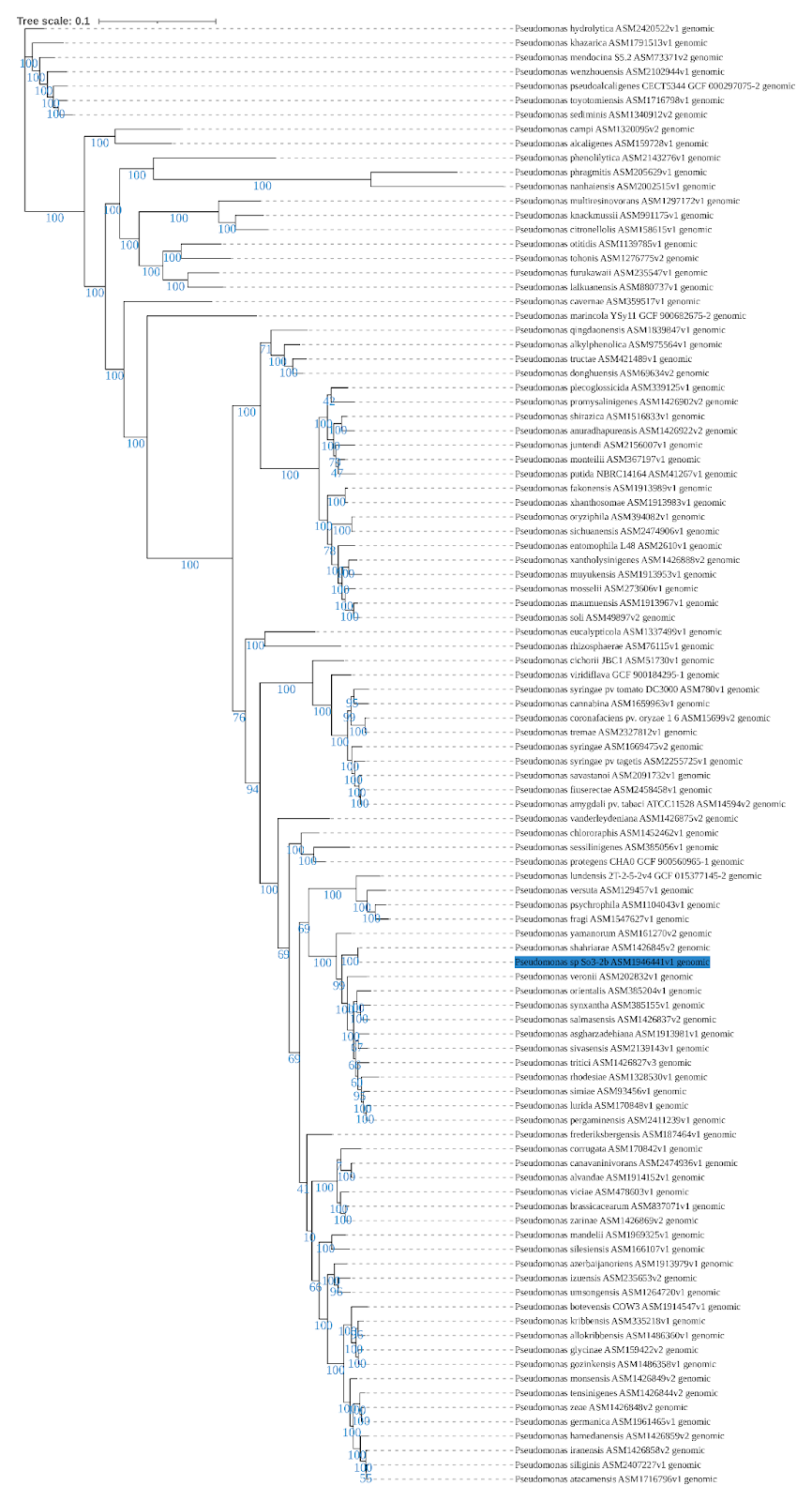


**Supplementary figure 3**. Unrooted maximum likelihood proteome phylogenetic tree, based on concatenated amino acids sequences from 152 ortholog genes present in 102 reference complete genomes from the *Pseudomonas* genus using RAxML. The NCBI accession number of each genome is present in the name. The branch lengths represent the number of substitutions per site (scale: 0.1 substitutions per site) and the percentage of the 1000 replications bootstraps is also shown. Antarctic strain So3.2b is marked in blue.
